# *Cryptosporidium* and *Giardia* in Livestock in Tigray, Northern Ethiopia and Associated Risk Factors for Infection: A Cross-Sectional Study

**DOI:** 10.3389/fvets.2021.825940

**Published:** 2022-01-14

**Authors:** Tsegabirhan Kifleyohannes, Ane Nødtvedt, John James Debenham, Getachew Terefe, Lucy J. Robertson

**Affiliations:** ^1^Department of Paraclinical Sciences, Faculty of Veterinary Medicine, Norwegian University of Life Sciences, Ås, Norway; ^2^College of Veterinary Medicine, Mekelle University, Mekelle, Ethiopia; ^3^Production Animal Clinical Sciences, Faculty of Veterinary Medicine, Norwegian University of Life Sciences, Ås, Norway; ^4^Department of Pathology and Parasitology, College of Veterinary Medicine and Agriculture, Addis Ababa University, Bishoftu, Ethiopia

**Keywords:** *Cryptosporidium*, Ethiopia, *Giardia*, livestock, risk factor

## Abstract

The occurrence and species/genotypes of *Cryptosporidium* and *Giardia duodenalis* infecting young livestock in selected districts of Tigray, Ethiopia were investigated, along with risks associated with infection. A total of 757 faecal samples were collected from calves, lambs, and goat kids from four rural districts in Tigray, and also from calves in periurban Mekelle, Tigray's main city, and analysed for *Cryptosporidium* oocysts and *Giardia* cysts. Farmers answered questionnaires regarding potential risk factors at sample collection. Immunofluorescent antibody staining was used for parasite detection, and PCR at selected genes and sequencing of positive samples was used for molecular characterisation. The occurrence of *Cryptosporidium* infection was 10, 9, and 4% in calves, lambs, and goat kids, respectively; equivalent figures for *Giardia* infection were 39, 32, and 21%. Molecular characterisation of *Cryptosporidium* isolates revealed *C. ubiquitum*, subtype XIIa in all three host species; *C. ryanae* in calves and goat kids; *C. andersoni* and *C. bovis* were identified only in calves, and *C. xiaoi* was identified in lambs. For *Giardia*, Assemblage E predominated in all host species, but among calf isolates we also identified a few potentially zoonotic genotypes (assemblages A (AI) and Assemblage B). Periparturient care was shown to be a particularly relevant risk factor for infection, and infections were less likely to occur under extensive management systems. Our major findings were widespread occurrence of both parasites in livestock, and the apparent lack of the most common zoonotic species. Our results are discussed in relation to other relevant studies. As our study was conducted in Tigray, further investigation in different settings in Ethiopia could provide relevant information on transmission and zoonotic potential. In addition, given the dependency on healthy animals for the livelihoods of the population of Tigray, investigation of the effect of these common parasites on livestock productivity is important.

## Introduction

In developing societies, the livelihoods of many people are dependent on livestock. In most parts of Africa, people and animals live in close proximity, and children have a substantial role in livestock-rearing activities (1). In low and middle-income countries, livestock, primarily cattle and small ruminants (sheep and goats), represent much more than food and nutrition (2) and are also used for income and employment, as well as a means of storing wealth. Livestock is also particularly valuable to women in Africa because it is among the few assets in many societies that women can own and manage themselves (3). Furthermore, livestock manure is used for fertiliser, as fuel for cooking, and for building materials, which are typically prepared by hand by women and children (4). However, this close association between people and their animals also provides the interface for the transmission of zoonotic pathogens (5).

Ethiopia has the largest livestock population in Africa. In Tigray, one of the regional states in northern Ethiopia, the livestock populations are estimated to be approximately 5 x 10^6^ cattle, 2 x 10^6^ sheep, and 5 x 10^6^ goats (6).

There are three main livestock production systems in Ethiopia: extensive, intensive, and semi-intensive (7). In Tigray, the livestock system is usually extensive, with animals mostly kept in small groups in simple pens close to the farmer's home (personal observation). These animals are often under the care of younger family members, and communal grazing is common (7). Drinking water for ruminant livestock in grazing areas is mainly provided from untreated sources (personal observation). Feeding strategies use naturally available forage (pasture and browse), with farmers using crop residues and hay as supplements during the dry season (7).

The protozoan parasites *Cryptosporidium* (Phylum Apicomplexa) and *Giardia* (Phylum Fornicata) are extremely common in ruminants, with worldwide distribution (8, 9). Both parasites can cause gastrointestinal disease in infected hosts, sometimes leading to considerable economic losses, and both also have zoonotic potential (8, 10).

*Cryptosporidium* infection is an important cause of clinical morbidity, mortality, and associated loss of production in ruminant livestock, particularly in young, especially neonatal, animals (8). Clinical signs of cryptosporidiosis in calves include pasty to watery diarrhoea, sometimes accompanied by lethargy, inappetence, fever, dehydration, and poor body condition. Similarly, neonatal lambs and goat kids with cryptosporidiosis have pasty to watery, yellow foul-smelling diarrhoea, anorexia, depression and abdominal pain (11). Infection in older animals is usually subclinical, but can still have a negative effect on production, with a lower body condition score, slower growth rate, and lower carcass weight and dressing percentage at slaughter (11).

Giardiasis in livestock is also characterised by diarrhoea, weight loss, and malabsorption. Infection may also be subclinical, but still affect growth and productivity (9). Chronic *Giardia* infections tend to occur toward the end of the neonatal period in ruminants, but their importance as a cause of diarrhoea is still ambiguous (8).

Over 30 species and over 70 genotypes of *Cryptosporidium* have been identified, some of which are host specific (12, 13). The four major *Cryptosporidium* spp. that infect cattle throughout the world are *C. parvum, C. bovis, C. ryanae*, and *C. andersoni*, of which *C. parvum* and *C. andersoni* are the two species that have been most associated with clinical disease in cattle (14). In sheep and goats, the most common species identified are *C. parvum, C. ubiquitum*, and *C. xiaoi*, and all three can be responsible for mild to severe diarrhoea and mortality (9, 11).

The zoonotic potential of *Cryptosporidium* depends on the species / genotype (11). *C. parvum* (subtypes IIa and IId) is the dominant zoonotic species in cattle and humans. Although information is scarce, some evidence suggests that sheep and goats can also be important as reservoirs for the zoonotic transmission of *Cryptosporidium*, particularly *C. parvum* (15, 16).

*Giardia duodenalis* is a species complex with eight distinct assemblages or genotypes, with some degree of host specificity (17–19). Globally, sub-assemblage AI is predominantly found in livestock and to a lesser extent in humans, whereas in most parts of the world, AII is mainly found in humans and a minor proportion in animals. In contrast, AIII has exclusively been found in animals, mainly in wildlife (17). Sub-assemblages AI, AII, BIII, and BIV are considered potentially zoonotic (17).

Among *Giardia* infections in domestic ruminants (cattle, goats, sheep) globally, including in Africa, Assemblage E is the predominant species reported and is not usually considered zoonotic (20, 21). However, Assemblages A (AI and AII) and B (BIV) have been reported in goats, cattle, and other animal species, both domesticated and wild, from various parts of Africa (18).

Although livestock infections with *Cryptosporidium* and *Giardia* have been reported from many parts of Ethiopia (Table 1), all published studies are from Addis Ababa and Oromia region (120 km distance from Addis Ababa) and very few studies included molecular characterisation (species and subtypes of *Cryptosporidium* or Assemblages of *Giardia*). The only published article on the molecular study of *Cryptosporidium* and *Giardia* in lambs from Addis Ababa and its surroundings reported *C. ubiquitum* and Assemblage E *Giardia* (21).

**Table 1:** Studies from Ethiopia on *Cryptosporidium* and *Giardia* infections in ruminant livestock, with detection methods and prevalence data.

**Region**	**Host animal**	**Age group**	**Number sampled**	**Detection method**	**Molecular characterisation**	**Prevalence (%)**	**References**
***Cryptosporidium*** **infections**							
Bishoftu (Debre Zeit) Oromia	Calves	<1 year	364	mZN	Not done	13.6	(22)
	Lambs	<1 year				16.9	
	Goat kids	<1 year				11.5	
Addis Ababa and Debre Zeit	Calves	<1 year	580	Sheather's flotation, mZN	Not done	17.6	(23)
North Shewa Zone, Oromia	Cattle	age not reported	384	mZN	Not done	7.8	(24)
Oromia special zone	Calves	<5 months	449	mZN PCR	*C. bovis,C. ryanae,C. andersoni*	15.8	(25)
Addis Ababa surrounding (Oromia special Zone)	Lambs	<5 months	389	mZN, PCR	*C. ubiquitum*	2.1	(21)
Addis Ababa and its surroundings	Calves	<2 months	392	mZN	Not done	17.3	(26)
		2–6 months				18.2	
		>6 months				21.5	
Holeta, Oromia	Dairy calves	<3 months	270	mZN, PCR	*C. parvum,C. andersoni*	14.8	(27)
Eastern Hararghe	Calves	<12 months	237	Sheather's flotation, mZN	Not done	27.8	(28)
	Lambs	<6 months				22.2	
	Goat kids	<6 months				12.2	
North West	Calves	<12 months	360	Sheather's flotation, mZN	Not done	18.6	(29)
Southern zone	Calves	<12 months	330	IFAT	Not done	13	(30)
***Giardia*** **infections**							
North Shewa Zone, Oromia	Cattle	age not reported	384	Direct wet mount with saline and iodine stain	Not done	2.3	(24)
Addis Ababa surrounding (Oromia special Zone)	Lambs	<5 months	389	Lugol's iodine PCR	Assemblage E	2.6	(21)
Southern zone	Calves	<12 months	330	IFAT	Not done	10	(30)

The epidemiology, impact, and zoonotic potential of these parasites in livestock in Ethiopia are therefore poorly understood. This is partly because most previous studies in livestock were based solely on light microscopy, using modified Ziehl-Neelsen (mZN) staining (23, 24). This method has poor sensitivity and specificity and cannot be used to identify species (and subtypes) or genotypes. Thus, knowledge on the epidemiology of *Cryptosporidium* and *Giardia* in young livestock in Ethiopia would be highly valuable, particularly from regions beyond the area in the vicinity of the capital, Addis Ababa, and also regarding zoonotic (public health) aspects.

This need underpinned the objectives of this study, which were specifically: i) to determine the occurrence of *Cryptosporidium* and *Giardia* infection in dairy calves, lambs, and goat kids in Tigray, Northern Ethiopia; ii) to identify the species (and subtypes) of *Cryptosporidium* and *Giardia duodenalis* genotypes in infected dairy calves, lambs, and goat kids, and thereby determine the zoonotic potential; and iii) to identify risk factors associated with the infection of livestock with these parasites.

## Materials and Methods

### Study Areas

The study was carried out between October 2018 and January 2019 in four districts of Tigray, Ethiopia; these are: Enderta (south east zone of the region), Kilte Awulaelo (east), Hintalo Wejirat (south east zone of the region), and Raya Azebo (south zone). Detailed information about these districts can be found in Kifleyohannes et al. (31). In addition, in February 2020, calf samples from urban farms were collected from Mekelle, the capital city of Tigray.

### Study Design and Sample Size Determination

A cross-sectional study design, based on a convenience sample rather than randomised sampling, was used to determine the occurrence in livestock from these four districts of Tigray by analysing faecal samples collected from calves, lambs, and goat kids. Sample size was determined by using expected prevalence of 14.8 % for *Cryptosporidium* and 2.3 % for *Giardia* infections in calves (24, 27), 16.9 % for *Cryptosporidium* and 2.6 % for *Giardia* infections in lambs (21, 22), and 11.5 % for *Cryptosporidium* in goat kids (22) using the formula given by Thrusfield (32). To calculate the sample size, 5 % absolute precision and 95 % confidence intervals were used for each species.

The calf samples from Mekelle were collected on an availability basis, with input from the city's animal production and health experts regarding where young calves were available for sampling within Mekelle.

### Study Animals

Animals were targeted through a door-to-door survey in all selected villages (tabia) at the specified study sites, and all homesteads within each village were contacted. Across the four districts, faecal samples were collected from 208 calves, 270 lambs, and 248 goat kids, all of which were ≤ 6 months of age and kept under intensive, semi-intensive, or extensive management systems. Of the calves, 65 (31%) were ≤ 8 weeks of age, and of these 21 (32%) were under 3 weeks of age.

For calves in Mekelle city, a total of 31 faecal samples were collected from 12 urban farms (two cooperative farms and 10 private farms). All calves were aged ≤ 3 months.

Information was recorded on animal age, sex, management system, faecal characteristics (consistency, colour, presence or absence of mucus), cleanliness of the pen and perianal area. For calves, the breed (local or crossbreed) was also recorded.

### Sample Collection and Analysis for *Cryptosporidium* Oocysts and *Giardia* Cysts

Approximately 5 g of faeces were collected directly from the rectum of each animal and placed into sampling bottles. The samples were preserved by mixing in 2.5 % potassium dichromate and then refrigerated at 4°C at the Parasitology Laboratory, at the College of Veterinary Medicine, Mekelle University (Mekelle, Ethiopia) before shipping to Norway. Potassium dichromate was removed from the faeces by repeated washings at the Parasitology Laboratory, Faculty of Veterinary Medicine, at the Norwegian University of Life Sciences (Oslo, Norway).

The subsamples were then homogenised, concentrated by centrifugation, and approximately 20 μl of the resulting pellet was placed on a microscope slide using a plastic loop. The samples were air-dried and then fixed with methanol before being analysed by using immunofluorescent antibody test (IFAT), with staining with 15 μl of fluorescein isothiocyanate (FITC)-labelled monoclonal antibody (Aqua-Glo, Waterborne Inc, NO, USA) for the detection of *Cryptosporidium* or *Giardia* (oo)cysts. After incubation of the samples and antibody at 37°C for 20 min, 10 μl of 4', 6' diamidino-2-phenylindole (DAPI) was added. After rinsing the samples, a drop of 1,4 diazabicyclo (2.2.2) octane (DABCO) anti-fade mounting medium was added and a coverslip placed over the sample. The stained samples were screened immediately after staining using a fluorescence microscope equipped with appropriate filters (FITC and DAPI) and Nomarski optics.

The entire slide was scanned under the FITC filter and, for presumptive (oo)cysts based on morphometry and staining, the morphology and intactness were checked by light microscopy with Nomarksi optics, as well as visualisation of DAPI staining of nuclei under the DAPI filter. A semi-quantitative measure of infection intensity was determined based on the number of cysts/oocysts per field of view at the objective 20x according to Utaaker, (33): +1 1–9 (oo)cysts, +2 10–50 (oo)cysts, +3 51–100 (oo)cysts, +4 >100 (oo)cysts.

### DNA Isolation

The samples containing higher numbers of (oo)cysts or where (oo)cysts were positive for DAPI (and therefore containing nuclei), were preferentially selected for DNA extraction. DNA was isolated using the DNeasy PowerSoil Kit protocol (Qiagen, Oslo, Norway), with slight modifications. The faecal pellet (250 μl) and 60 μl of the lysis solution (solution C1) were added to the PowerBead Tubes and vortexed together to mix. This was then subjected to bead beating to release the DNA by breaking the (oo)cysts walls using a FastPrep-24 5G (MP Biomedicals) in two cycles of 4 m/s for 60 s with a 45 s pause between the cycles. Finally, the DNA was eluted in 50 μl of the elution solution (solution C6) and stored at −20°C. Studies by Elwin et al. (34, 35) reported that the bead-beating approach is effective for obtaining the DNA of *Cryptosporidium* and *Giardia* from human stools.

### Polymerase Chain Reaction and Sequencing

For samples positive for *Cryptosporidium*, primers targeting sections of the SSU-rRNA gene and gp60 gene for *C. ubiquitum* were used for sequence investigation by conventional PCR. Four genes were targeted for genotyping investigations of the *Giardia*-positive samples: glutamate dehydrogenase (GDH) gene, beta-giardin (BG) gene, triosephosphate isomerase (TPI) gene, and SSU (rRNA) gene. In addition, three genes were used to subtyping Assemblage A samples: DNA repair and recombination gene (RHP26), high cysteine membrane gene (HCMP), and NEK kinase gene. Assemblage B subtyping were performed using the following three genes, 6-phosphogluconate dehydrogenase (pdg), Hypothetical protein, and Phosphorylase B kinase gamma catalytic chain (phkg2). Primers and reaction cycles, as well as other details, for all PCR used in this study are provided in Supplementary File 1.

The PCR products were examined after separation on a 2% agarose gel, stained with SYBERsafe DNA gel stain and visualised under UV illumination. A 100 bp ready-to-use DNA ladder (Thermo Scientific) was used for fragment size determination. Purification of the positive products was carried out using either ExoSAP-IT PCR product clean-up reagent (Thermofisher Scientific) or PureLink Quick Gel extraction and PCR purification Combo kit (Thermo Fisher Scientific) and sent to a commercial company (Eurofins Genomics, Germany) for sequencing in both directions. Sequences were checked using Geneious Prime software, contigs made, and compared with sequences already deposited in GenBank using the Basic Local Alignment Search Tool (NCBI BLAST), along with a recently developed online tool, SSU and gp60 Cryptogenotyper for *Cryptosporidium* sequences (36).

### Questionnaire Survey

A pre-tested questionnaire was used to collect data on the study animals, their owners, and management practises. The questionnaires were filled out during individual interviews with the animal owners by the first author, supported by colleagues from the College of Veterinary Medicine of Mekelle University, along with veterinarians and technicians at the study districts. Data on risk factors of *Cryptosporidium* and *Giardia* infections such as age, feeding method, source of drinking water, the occurrence of diarrhoea, pen cleanliness, etc. were collected (see Supplementary File 2 for questionnaire). The questionnaires were designed to contain mainly closed-ended questions.

### Statistics

A database was created in Excel and analysed using STATA 15 software (STATA SE Corp, TX, USA) software. The Chi-square test and Fisher's exact test were used to evaluate the association between risk factors for *Cryptosporidium* and *Giardia* infection. Variables significant at *P* < 0.05 in the above tests were considered as candidates for a multivariable model. Subsequently, a backward stepwise logistic regression was used to identify possible predictors of the *Cryptosporidium* and *Giardia* infection out of the following variables: districts, periparturient care, weaning age, management system and breed. At each step, the variable with the highest *p*-value in the multivariable logistic regression model was removed before re-running the multivariable logistic regression model and these iterations of elimination were continued as long as the values of the Akaike information criterion (AIC) of the new models were decreasing. The confidence level was maintained at 95 % and *P* < 0.05 was considered for the significance level.

## Results

### Overview of *Cryptosporidium* and *Giardia* Infections in Calves, Lambs, and Goat Kids From the Four Districts

The overall combined occurrence of *Cryptosporidium* spp. and *Giardia* spp. in the four districts in all livestock (calves, lambs, and goat kids) were 8 % (56/726) and 30 % (220/726), respectively. Investigation of associations between the occurrence of infection and livestock species showed statistically significant differences for both *Cryptosporidium* (*P* = 0.024) and *Giardia* (*P* = 0.001). The highest occurrence of *Cryptosporidium* was observed in calves and the lowest in goat kids, and the same pattern was found with *Giardia*.

Statistically significant differences (*P* = 0.015) were found between the occurrences of *Cryptosporidium* in the four districts: the highest occurrence was in Enderta (11 %) and the lowest occurrence was found in Hintalo Wejirat (3 %). No statistically significant difference was observed between the presence of *Giardia* in the four districts.

**Figure 1:**
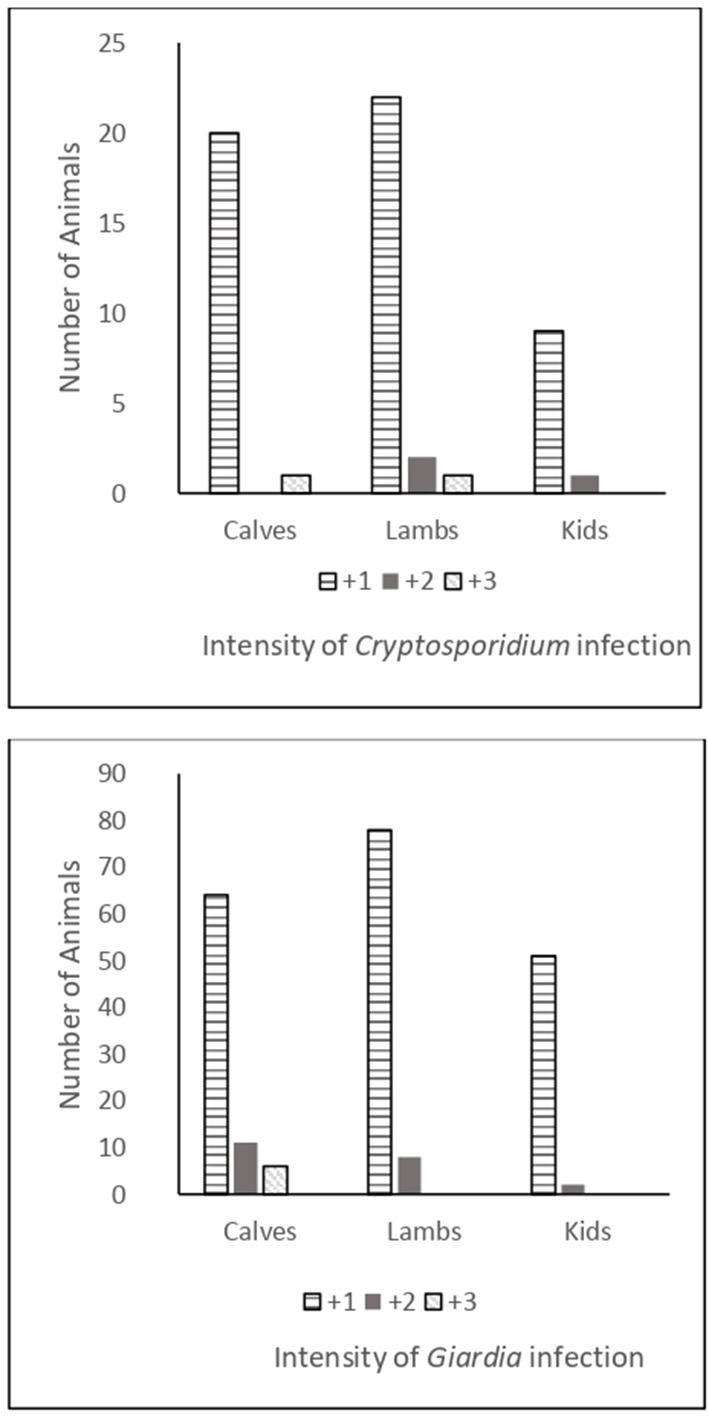
Intensity of *Cryptosporidium* and *Giardia* infections in calves, lambs, and goat kids.

The intensity of *Cryptosporidium* infection between livestock species was as shown in Figure 1, with most infected animals shedding relatively low numbers of oocysts (+1). The intensity of infection in *Giardia*-infected animals varied between livestock species. Although most infected animals shed only low quantities of cysts (+1), relatively high *Giardia*-cyst counts were recorded in several calves.

The occurrence of *Giardia* infection among the three species of the study animals was compared in different age groups of animals using the chi-square test. Calves and lambs ≤ 8-weeks were more likely to have *Giardia* infection than calves and lambs of >8 weeks, whereas the opposite was seen for goat kids; goat kids over 8 weeks were more likely to be infected with *Giardia* than goat kids of 8 weeks and younger (Table 2). Although *Cryptosporidium* infections tended to occur more often within the younger age group for all livestock species, no clear age-related associations were identified.

**Table 2:** Comparison of the occurrences of *Cryptosporidium* and *Giardia* infections between different age groups of calves, lambs, and goat kids.

**Animals**	**Age**	**Total**	***Cryptosporidium***			***Giardia***		
			**Positives**	**Proportion (%)**	***P*-value**	**Positives**	**Proportion (%)**	***P*-value**
Calves	≤ 8 weeks	65	9	14	0.226	32	49	0.040
	> 8 weeks	143	12	8		49	34	
Lambs	≤ 8 weeks	78	8	10	0.738	33	42	0.022
	> 8 weeks	190	17	9		53	28	
Goat Kids	≤ 8 weeks	65	4	6	0.303	8	12	0.041
	> 8 weeks	185	6	3		45	24	

### *Cryptosporidium* and *Giardia* Infections in Calves by District and Different Management Systems

The Chi-square test showed a significant difference in *Cryptosporidium* infection in calves in all four districts. The occurrence of infection in the calves of Kilte Awulaelo district (21%) was significantly higher than that in the other districts; see Supplementary File 3 (Supplementary Table 1). The occurrence of infection was significantly higher in crossbreed calves (33 %) than in local breed calves (7%); see Supplementary File 3 (Supplementary Table 1).

However, when multivariable logistic regression analysis was used, only management system and periparturient care were found to be significant. Calves in an intensive management system were more likely to be infected with *Cryptosporidium* than calves in an extensive management system (OR = 7.8, 95% CI = 2.46–24.49: *P* = 0.001). Calves that stayed in the same barn with cows after birth were at higher risk of *Cryptosporidium* infection than calves that stayed in their pen alone or with other calves (OR = 3.2, 95% CI = 1.02–10.08; *P* = 0.04).

The occurrence of *Giardia* infection in calves was similar among the four districts and with respect to their periparturient care, weaning age, type of feed supplement, type of management system, and breeds see Supplementary File 3 (Supplementary Table 2).

In calves in urban farms in Mekelle city, out of 31 calf faecal samples collected from 12 urban farms, 13% contained *Cryptosporidium* spp. oocysts and 35% contained *Giardia* cysts.

### Molecular Characterisation of *Cryptosporidium* and *Giardia* From Calf Infections

Of the 21 *Cryptosporidium-*positive samples from the four districts, species determination was attempted on 15, but only nine were successfully amplified and sequenced. Additionally, of the four *Cryptosporidium-*positive calf samples from Mekelle city, molecular characterisation was successful for three. Accordingly, *C. andersoni* (*n* = 1), *C. bovis* (*n* = 3), *C. ryanae* (*n* = 7), and *C. ubiquitum* (*n* = 1) were identified from the four districts, whereas only *C. andersoni* and *C. ryanae* were identified in the urban farms. The *C. ubiquitum* sample was subtyped and found to belong to the XIIa family.

Of the 81 *Giardia*-positive calf samples from the four districts, 31 were selected for molecular characterisation, of which 18 provided good sequence results; 14 were Assemblage E, three were Assemblage A, and one was Assemblage B. Subtyping the Assemblage A isolates revealed that two were AI and one could not be subtyped. Subtyping of the Assemblage B using multilocus sequence typing showed a close relationship with Algerian samples (37). Out of 11 *Giardia*-positive calf samples from urban farms, molecular characterisation was successful for six and all were Assemblage E.

Representative sequences have been deposited in GenBank and can be found under Accession numbers OK336067, OK336072-OK336079, OK336082-83 and OK358701-OK358702 (*Cryptosporidium*) and OK523961-OK523964, OK523969-OK523975, OK626272-OK626274, OK626276-OK626277 (*Giardia*).

### *Cryptosporidium* Infection in Lambs and Goat Kids by District and Different Management Systems

Based on the Chi-square test, the occurrence of *Cryptosporidium* infection in lambs showed significant differences among the four districts. The occurrence of infection in lambs in the Enderta district (16%) was significantly higher than in the other districts; see Supplementary File 3 (Supplementary Table 3). However, such differences were not observed in goat kids among different districts. The only significant difference was that the occurrence of *Cryptosporidium* in goat kids housed with adults and other animals (9%) was higher than in goat kids kept with does in their pen (1.3%) see Supplementary File 3 (Supplementary Table 4). Lambs kept under a semi-intensive management system had a significantly higher occurrence of *Cryptosporidium* infection (19%) than lambs reared under an extensive management system (7%); see Supplementary File 3 (Supplementary Table 3).

However, analysis using multivariable logistic regression only showed a significantly higher occurrence of *Cryptosporidium* infection in lambs housed with adults and other animals than lambs kept with ewes in their pen (OR = 3.7, 95% CI = 1.12–11.19; *P* = 0.020). Lambs from Enderta were more likely to be infected with *Cryptosporidium* infection than lambs from Kilte Awulaelo and Hintalo Wejirat (OR = 5.1, 95% CI =1.07–23.88; *P* = 0.040). Lambs from Raya Azebo were more likely to be infected with *Cryptosporidium* infection than lambs from Kilte Awulaelo and Hintalo Wejirat (OR = 6.1, 95% CI = 1.14–32.4; *P* = 0.035).

### *Giardia* Infection in Lambs and Goat Kids by District and Different Management Systems

Analysis using Chi-square test showed that the occurrence of *Giardia* infection in lambs was similar among the four districts, and not affected by age at colostrum feeding and type of feed supplement. However, lambs, but not goat kids, kept under semi-intensive management had a significantly higher occurrence of *Giardia* infection (44%) than lambs reared in extensive management (29%); see Supplementary File 3 (Supplementary Tables 5, 6). Multivariable logistic regression revealed that lambs housed with ewes and other animals were more likely to be infected with *Giardia* than lambs kept with ewes in their pen (OR = 2.7, 95% C = 1.42–5.21; *P* = 0.002).

### Molecular Characterisation of *Cryptosporidium* and *Giardia* From Lamb Infections

Out of 93 lamb faecal samples positive for *Cryptosporidium* oocysts and/or *Giardia* cysts, DNA was extracted from 20. Of the 25 *Cryptosporidium-*positive lamb samples, only seven were selected for molecular characterisation. For five of them, amplification was successful and resulted in good sequences; three were identified as *C. ubiquitum* and two as *C. xiaoi*. All three *C. ubiquitum* isolates were subtyped as belonging to the XIIa family.

Of the 86 *Giardia*-positive lamb samples, most were very light infections containing few cysts; however, 17 were selected for molecular characterisation, and amplification and sequencing were successful for 12 of these. All the samples were found to be Assemblage E.

Representative sequences have been deposited in GenBank and can be found under Accession numbers OK336068-OK336070, OK336084-OK336087, OK358703-OK358704 (*Cryptosporidium*) and OK523965-OK523966, OK523976, OK626275 (*Giardia*).

### Molecular Characterisation of *Cryptosporidium* and *Giardia* From Goat Kid Infections

Out of 61 goat kid faecal samples positive for *Cryptosporidium* oocysts and/or *Giardia* cysts, DNA was extracted from 11. Of the three *Cryptosporidium-*positive goat-kid samples selected for molecular characterisation, DNA from two of them was amplified and sequenced successfully and revealed *C. ryanae* and *C. ubiquitum*. The latter was subsequently identified as the XIIa subtype.

Of 10 *Giardia*-positive goat kids samples selected for molecular characterisation, only six samples provided good sequence results at one or more genes; all were identified as Assemblage E.

Representative sequences have been deposited in GenBank and can be found under Accession numbers OK336071, OK336080-81 and OK358705-06 (*Cryptosporidium*) and OK523967-OK523968, OK523977-OK523978 (*Giardia*).

## Discussion

### *Cryptosporidium* and *Giardia* Infections in Calves, Lambs, and Goat Kids

The main finding of this study is that both parasites occur widely and frequently within livestock in rural Tigray, with a significantly higher occurrence in calves than small ruminants, particularly goat kids. The low infection in goat kids may reflect that they are browsers, and thus the likelihood of ingesting the transmission stages of these parasites from the ground is lower than for the other grazing species (38). Another important finding was that in the four study districts, *Cryptosporidium* infection of livestock occurred significantly less frequently than *Giardia* infection and a similar pattern was seen among calves from the urban farms in Mekelle. However, for calves, more variables had significant association with *Cryptosporidium* infection than with *Giardia* infection. This might be attributed to the robustness of *Cryptosporidium* and survival capability to stay in the environment and contaminate the barn. In addition, in the study areas, although intensive management and cross breeds were reported by some farmers concerning calves, for lambs and goat kids only local breeds were reported, with extensive management almost exclusively predominating, and intensive management not reported at all. This is in line with a report by Vermeulen et al. (39), who found that intensively managed calves are the dominant source of oocysts compared with other livestock species. Although *Giardia* cysts are robust and can survive well in aquatic environments, they are relatively fragile compared with *Cryptosporidium* oocysts and are unlikely to survive for prolonged periods in the animal houses (40).

Unlike in our study, other studies of livestock in Ethiopia for both parasites have tended to find higher prevalences of *Cryptosporidium* infection than *Giardia* (see Table 1). However, most other studies focused only on *Cryptosporidium*, and generally report higher prevalences than found in our investigation. Differences in occurrence for both parasites should be expected between studies, due to variations in management practises, transmission possibilities, host susceptibilities, and other parameters. Of particular relevance may be that most of the samples in our study were from extensively managed animals, but in some of the other studies from Ethiopia the animals were intensively managed [e.g., 22, 28]. Intensive management may promote transmission, due to closer contact between infected animals and build-up of oocyst contamination within a restricted environment.

However, it also seems likely that methodological specificities and sensitivities may be of relevance. Most other studies from Ethiopia used mZN for detection of *Cryptosporidium* (21, 24, 27), which is less sensitive and less specific than the IFAT technique used in our study. A study from Tanzania on *Cryptosporidium* in calves (41) found that misidentification occurred when mZN was used, and commented that false-positive results may occur when mZN is the sole detection method used.

There are only three published reports from Ethiopia on the prevalence of *Giardia* in livestock (Table 1), and our results describe a considerably higher prevalence than all the previous studies. Methodological differences may also play a role here, as a less sensitive method (light microscopy with Lugol's iodine staining) was used in one study for identifying *Giardia* infection in lambs (21). However, another study that investigated *Giardia* shedding in calves in southern Ethiopia (30) used IFAT, but also found a much lower prevalence (10%; see Table 1).

Although both infections were found in all species of livestock in all districts investigated, and occurrences were similar for *Giardia* among districts, for *Cryptosporidium* the occurrences varied significantly according to district. That we found the highest occurrence of *Cryptosporidium* infection in Enderta followed by Kilte Awulaelo, is interesting as surveys of fresh produce and water in all four districts found contamination with these parasites in these two districts only (31, 42). This might suggest that this parasite is circulating more successfully among animals and in the environment in these two districts.

Most animals included in our study had relatively low intensities of infection. Most studies from different parts of the world do not report the intensity of infection. The low levels of intensity of infection found in our study may reflect that the majority of samples were collected from extensive management systems where trickle infections, with concomitant build-up of immunity, may be more likely to occur than abrupt exposure to a massive infectious dose.

The prevalence of *Cryptosporidium* infection was similar in the different age groups of animals investigated in our study, although there was a trend that the occurrence was higher within the younger age group for all livestock species. In a report from Nigeria, young calves (<3 months old) had higher infection rates of *Cryptosporidium* than calves > 3 months old (43).

The occurrence of *Giardia* infection was higher in calves and lambs ≤ 8weeks old than older calves and lambs suggesting that the infection rates decline as the age of the animals increases. Similar results have been reported in other countries. In Canada and China the prevalence of *Giardia* infection was greater in calves and lambs than adults (44, 45) and this is mostly consistent with our findings. However, we found that *Giardia* infection in goat kids was significantly higher in the age group > 8 weeks than ≤ 8 weeks old.

Compared with *Giardia* cyst excretion from calves and lambs, relatively few cysts were found in most *Giardia*-positive goat kids. This low shedding of *Giardia* cysts from infected goat kids might be related to goats being selective browsers, and therefore less exposed to infectious cysts.

### Risk Factors for *Cryptosporidium* and *Giardia* Infections in Calves, Lambs, and Goat Kids

We found that *Cryptosporidium* infection occurred more often in crossbreed calves than local breeds. The potential effect of cattle breed on *Cryptosporidium* infection has been previously discussed (13). In a study from Malaysia (46), it was found that Mafriwal cattle (Sahiwal × Friesian crosses) and Jersey × Friesian crosses had higher rates of infection. This could also be due to the different management system, as crossbreed calves tended to be intensively managed compared with local breeds. Higher rates of *Cryptosporidium* infection in lambs under semi-intensive systems probably reflects the relatively high confinement in semi-intensive management, which results in greater crowding and a more contaminated environment than in farms using an extensive system. No significant difference was observed in goat kids under both management systems in our study.

Calves housed with cows had a significantly higher *Cryptosporidium* infection than calves housed in their pen alone or with other calves in our study; this finding is similar to those reported by Manyazewal et al. (27). This strong association between periparturient care and *Cryptosporidium* infection in calves is probably associated with the level of hygiene when calves are kept alone or with other animals. This suggestion is supported by Abebe et al. (23), who reported a substantial connexion between *Cryptosporidium* infection and the hygienic status of dairy animals and their farms. Similarly, Ayele et al. (29) stated that poor hygiene increases the infection rate and spread of *Cryptosporidium* among animals. This is probably because dirty and muddy farms may create favourable conditions for the presence or survival of *Cryptosporidium* oocysts. This increases the likelihood of feed and water contamination, which, in turn, might favour exposure of calves to *Cryptosporidium* oocysts. We found similar results for ewes and lambs and for goat kids and does housed together in a single pen, and, again, a likely explanation is close contact between the animals and contamination, along with less exposure to inactivation pressures such as desiccation and UV-irradiation (13).

### Molecular Characterisation of *Cryptosporidium* and *Giardia* in Calves, Lambs, and Goat Kids

Perhaps the most important finding of this cross-sectional study is that, despite the close contact between animals and humans, the main zoonotic species and subtypes of *Cryptosporidium* and *Giardia* were not detected in any of the animal samples. However, species such as *C. andersoni* and *C. ubiquitum*, which are usually considered to have limited zoonotic potential (47, 48), were detected. Similarly, the *Giardia* assemblages identified were mostly non-zoonotic Assemblage E, but a few *Giardia* isolates detected in calves were of the potentially zoonotic Assemblages A and B.

Globally, four species of *Cryptosporidium* occur commonly in cattle: *C. parvum, C. bovis, C. ryanae* and *C. andersoni* (14). However, the majority of studies from Africa have indicated that *C. parvum* occurs relatively infrequently in cattle here (13). Nevertheless, one study from Egypt reported all the common *Cryptosporidium* spp., except for *C. andersoni* (49), and, in Ethiopia, all four common species have been reported in calves from the Oromia region by Wegayehu et al. (25) and Manyazewal et al. (27). Our findings seem more similar to those from a study from Nigeria that reported all species of *Cryptosporidium* from calves, except *C. parvum* (43). It could be argued that *C. parvum* was not detected in calves in our study, as it is more common in very young calves. Of the 65 calves ≤ 8 weeks old included in our study, the age range was from 4 days, with 21 ≤ 3 weeks of age. Interestingly, our findings also differ from other reports from Africa, as we also detected *C. ubiquitum*, subtype XIIa in cattle. *C. ubiquitum* is considered an emerging zoonotic pathogen, with a wide geographic distribution and broad host range (47). However, to the best of our knowledge, there is only a single report of human infection with *C. ubiquitum* in Africa, with just one child from Nigeria reported to be infected with *C. ubiquitum* (then described as cervine genotype) among a survey of 692 children, of whom 134 were considered to be infected with *Cryptosporidium* (50). Thus, it appears that zoonotic transmission of this species of *Cryptosporidium* is not well established in Africa; reasons for this are discussed in the review article of Robertson et al. (13).

Globally, the most common species of *Cryptosporidium* identified in sheep are *C. ubiquitum* and *C. xiaoi*, although in some European countries *C. parvum* is also prevalent in sheep (51). The report by Robertson et al. (13), that *C. parvum* occurs relatively infrequently in African small ruminants, with *C. xiaoi* and *C. ubiquitum* predominating, is in line with our findings.

In goats, the most common *Cryptosporidium* spp. identified from different parts of the world are *C. ubiquitum, C. xiaoi*, and *C. parvum* (9, 52, 53). However, in our study only *C. ryanae* and *C. ubiquitum* were detected in goat kids. A recent review of *Cryptosporidium* in goats (38), reported that in studies from different parts of the world (Europe, Asia, Africa, South America, and Oceania) where *C. ubiquitum* has been reported, only subtype-XIIa has been detected (where subtyping has been conducted) and we also found this sub-type.

The *Giardia* assemblages identified in this study indicated that calves mainly carried Assemblage E infections and all *Giardia* isolates from lambs and goat kids belonged to Assemblage E. This Assemblage, which is generally not considered zoonotic, is usually found in cattle, sheep, goats, and pigs (9, 17). However, we did identify a few potentially zoonotic Assemblages in calves, with three Assemblage A isolates and one Assemblage B isolate, and consistent results among the different genetic loci amplified by PCR. Our finding of Assemblage B in calves is rather unusual, but Sprong et al. (17) also noted a very low percentage of Assemblage B in cattle. Our case could, potentially, indicate carriage rather than infection. Subtyping of two Assemblage A isolates revealed AI, which is mainly found in animals (17).

## Conclusion

Our study found a widespread occurrence of *Cryptosporidium* and *Giardia* in livestock in Tigray, Ethiopia. Five *Cryptosporidium* species, namely *C. bovis, C. ryanae, C. andersoni, C. xiaoi*, and *C. ubiquitum*, were identified in calves, lambs, and goat kids, with *C. ryanae* and *C, ubiquitum* being the predominant species. The zoonotic species, *C. parvum*, was not detected in any of the animal samples. Similarly, the majority of the *Giardia* assemblages detected in the samples were Assemblage E.

Periparturient care seemed to be particularly important regarding these infections in livestock, with crowding among calves, lambs, and goat kids, particularly mixing with adults and/or other animals, increasing the vulnerability to infection with both these parasites. Similarly, if the management system was more intensive, then the likelihood of infection was greater.

Conducting further research from different locations and settings in Ethiopia could provide relevant information regarding transmission dynamics for both parasites and the potential for zoonotic transmission. It would also be important to ascertain the effect that these parasites have on the productivity of the livestock, given that the farmers are dependent on healthy animals for their livelihoods.

## Data Availability Statement

The datasets presented in this study can be found in online repositories. The names of the repository/repositories and accession number(s) can be found at: https://www.ncbi.nlm.nih.gov/genbank/, OK336067, OK336072-OK336079, OK336082-83, OK358701-OK358702; OK523961-OK523964, OK523969-OK523975, OK626272-OK626274, OK626276-OK626277, OK336068-OK336070, OK336084-OK336087, OK358703-OK358704, OK523965-OK523966, OK523976, OK626275, OK336071, OK336080-81, OK358705-06, OK523967-OK523968 and OK523977-OK523978.

## Ethics Statement

The animal study was reviewed and approved by the Regional Committee for Medical and Health Research Ethics, South East; REK, SE, case number 2018/1279 C and the National Research Ethics Review Committee in Ethiopia (Ref. No: MoSHE/RD/144/1095/19). Written informed consent was obtained from the owners for the participation of their animals in this study.

## Author Contributions

TK, AN, JD, GT, and LR: conceptualization, methodology, and writing-review and editing. LR: validation. TK, AN, JD, and LR: formal analysis. TK: investigation, data curation, and writing original draft preparation. TK and LR: visualization and funding acquisition. LR, AN, JD, and GT: supervision. LR: project administration. All authors have read and agreed to the published version of the manuscript.

## Funding

This study was funded by the Norwegian Ministry of Foreign Affairs through the MU-NMBU Institutional Collaboration Phase IV, Improved Livelihood of the Rural Communities in the Arid Highlands of Northern Ethiopia.

## Conflict of Interest

The authors declare that the research was conducted in the absence of any commercial or financial relationships that could be construed as a potential conflict of interest.

## Publisher's Note

All claims expressed in this article are solely those of the authors and do not necessarily represent those of their affiliated organizations, or those of the publisher, the editors and the reviewers. Any product that may be evaluated in this article, or claim that may be made by its manufacturer, is not guaranteed or endorsed by the publisher.
