# Supplementary material for: Cryptosporidium and Giardia in Livestock in Tigray, Northern Ethiopia and Associated Risk Factors for Infection: A Cross-Sectional Study
Source: Front Vet Sci. 2022 Jan 14;8:825940. doi: 10.3389/fvets.2021.825940 (PMC8795829; doi:10.3389/fvets.2021.825940)
Supplement: Supplementary file 1 [file Data_Sheet_1.docx]

| **PCR conditions and primers** |  |  | | | | |  |
| --- | --- | --- | --- | --- | --- | --- | --- |
| **Target genes** | **Primers** | **Cycling conditions** | | | | | **References** |
| ***Cryptosporidium: s*mall subunit (SSU) rRNA** | | | | | | |  |
| First amplification,  Amplicon size: 1325 bp | SSU F1: (5’ TTCTAGAGCTAATACATGCG-3’)  SSU R1: (5’-GACGAGCCTCTGCACAACATC-3’) | | 95°C, 3 min | | | | Jiang et al., 2005 |
|  |  |  | 94°C, 45 sec | |  | |  |
|  |  |  | 55°C, 45 sec | | 35x | |  |
|  |  |  | 72 °C, 60 sec | |  | |  |
|  |  |  | 72 °C, 7 min | | | |  |
| Second amplification  Amplicon size: 840 bp | SSU F2: (5’ GGAAGGGTTGTATTTATTAGATAAAG-3’)  SSU R2: (5’-CTCATAAGGTGCTGAAGGAGTA-3’) | | 95°C, 3 min | | | | Jiang et al., 2005 |
|  |  |  | 94°C, 45 sec |  | | |  |
|  |  |  | 55 °C, 45 sec | 35x | | |  |
|  |  |  | 72 °C, 60 sec |  | | |  |
|  |  |  | 72 °C, 7 min |  | | |  |
| ***Cryptosporidium ubiquitum: 60-kDa glycoprotein*** | | | | | | | Li et al., 2014  Li et al., 2014 |
| First amplification,  Amplicon size: 1044 bp | F: (5’- TTTACCCACACATCTGTAGCGTCG -3’) | | 95°C, 3 min | | | |  |
|  | R: (5’- ACGGACGGAATGATGTATCTGA-3’) | | 94°C, 45 sec | | |  |  |
|  |  | | 58°C, 45 sec | | | 35x |  |
|  |  | | 72 °C,60 sec | | |  |  |
|  |  | | 72 °C, 7 min | | | |  |
| Second amplification  Amplicon size: 948 bp | F: (5’- ATAGGTGATAATTAGTCAGTCTTTAAT-3’) | | 95°C, 3 min | | | |  |
|  | R: (5’- TCCAAAAGCGGCTGAGTCAGCATC-3’) | | 94°C, 45 sec | | |  |  |
|  |  | | 55°C, 45 sec | | | 35x |  |
|  |  | | 72 °C, 60 sec | | |  |  |
|  |  | | 72 °C, 7 min | | | |  |
| ***Giardia: s*mall subunit (SSU) rRNA** | | | | | | | Read et al., 2002 |
| First amplification  Amplicon size: 292 bp | F: RH11(5’-CATCCGGTCGATCCTGCC-3’) | | 95°C, 3 min | | | |  |
|  | R: RH4 (5’-AGTCGAACCCTGATTCTCCGCCAGG-3’) | | 96°C, 30 sec | | |  |  |
|  |  | | 59°C, 40 sec | | | 40x |  |
|  |  | | 72 °C, 40 sec | | |  |  |
|  |  | | 72 °C, 7 min | | | |  |
| Second amplification  Amplicon size: 175 bp | F: GiarF (5’-GACGCTCTCCCCAAGGAC-3’) | | 95°C, 3 min | | | |  |
|  | R: GiarR (5’-CTGCGTCACGCTGCTCG-3’) | | 96°C, 30 sec | | |  |  |
|  |  | | 55°C, 40 sec | | | 40x |  |
|  |  | | 72 °C, 30 sec | | |  |  |
|  |  | | 72 °C, 7 min | | | |  |
| ***Giardia*: glutamate dehydrogenase (GDH)** | | | | | | | Cacciò et al., 2008 |
| First amplification  Amplicon size: 755 bp | F: GDH1 (5’-TTCCGTRTYCAGTACAACTC-3’) | | 95°C, 3 min | | | |  |
|  | R: GDH2 (5’-ACCTCGTTCTGRGTGGCGCA-3’) | | 94°C, 30 sec | | |  |  |
|  |  | | 50°C, 30 sec | | | 35x |  |
|  |  | | 72 °C, 60 sec | | |  |  |
|  |  | | 72 °C, 7 min | | | |  |
| Second amplification  Amplicon size: 530 bp | F: GDH3 (5’-ATGACYGAGCTYCAGAGGCACGT-3’) | | 95°C, 3 min | | | |  |
|  | R: GDH4 (5’- GTGGCGCARGGCATGATGCA -3’) | | 94°C, 45 sec | | |  |  |
|  |  | | 54°C, 45 sec | | | 40x |  |
|  |  | | 72 °C, 45 sec | | |  |  |
|  |  | | 72 °C, 7 min | | | |  |
| ***Giardia*: beta giardin (BG)** | | | | | | | Lalle et al., 2005 |
| First amplification  Amplicon size: 753 bp | F: G7(5’-AAGCCCGACGACCTCACCCGCAGTGC-3’) | | 95°C, 3 min | | | |  |
|  | R: G759 (5’-GAGGCCGCCCTGGATCTTCGAGACGAC-3’) | | 94°C, 30 sec | | |  |  |
|  |  | | 60°C, 30 sec | | | 35x |  |
|  |  | | 72 °C, 60 sec | | |  |  |
|  |  | | 72 °C, 10 min | | | |  |
| Second amplification  Amplicon size: 511 bp | F: (5’- GAACGAGATCGAGGTCCG-3’) | | 95°C, 3 min | | | |  |
|  | R: (5’- CTCGACGAGCTTCGTGTT-3’) | | 95°C, 30 sec | | |  |  |
|  |  | | 53°C, 30 sec | | | 40x |  |
|  |  | | 72 °C, 60 sec | | |  |  |
|  |  | | 72 °C, 10 min | | | |  |
| ***Giardia:* Triosephosphate isomerase (TPI)** | | | | | | | Sulaiman et al., 2003 |
| First amplification  Amplicon size: 605 bp | F: (5’-AAATYATGCCTGCTCGTCG-3’)  R: (5’-CAAACCTTYTCCGCAAACC-3’) | | 95°C, 3 min | | | |  |
|  |  |  | 94°C, 45 sec | | |  |  |
|  |  |  | 50°C, 45 sec | | | 35x |  |
|  |  |  | 72 °C, 60 sec | | |  |  |
|  |  |  | 72 °C, 10 min | | | |  |
| Second amplification,  Amplicon size: 530 bp | F: (5’- CCCTTCATCGGIGGTAACTT -3’)  R: (5’- GTGGCCACCACICCCGTGCC-3’) | | 95°C, 3 min | | | | Sulaiman et al., 2003 |
|  |  |  | 94°C, 45 sec | | |  |  |
|  |  |  | 50°C, 45 sec | | | 35x |  |
|  |  |  | 72 °C, 60 sec | | |  |  |
|  |  |  | 72 °C, 10 min | | | |  |
| ***Giardia***: **NIMA-related kinase (**NEK) 15411 | | | | | | |  |
| First amplification  Amplicon size: 902 bp | F: (5’- TCTCCTCAACGACCCCTCAATC -3’)  R: (5’- CCGAGTCTACCTCACCACACTCAC-3’) | | 95°C, 3 min | | | | Ankarklev et al., 2018 |
|  |  |  | 95°C, 20 sec | |  | |  |
|  |  |  | 55°C, 30 sec | | 35x | |  |
|  |  |  | 72 °C, 45 sec | |  | |  |
|  |  |  | 72 °C, 7 min | | | |  |
| Second amplification  Amplicon size: 700 bp | F: (5’- GATGGCAATCGGCTTCTCC -3’)  R: (5’- CCGAGTCTACCTCACCACACTCAC-3’) | | 95°C, 3 min | | | | Ankarklev et al., 2018 |
|  |  |  | 95°C, 20 sec |  | | |  |
|  |  |  | 55°C, 30 sec | 35x | | |  |
|  |  |  | 72 °C, 45 sec |  | | |  |
|  |  |  | 72 °C, 7 min | | | |  |
| ***Giardia:* DNA repair and recombination protein RHP 26** | | | | | | |  |
| First amplification  Amplicon size: 864 bp | F: (5’- GGTCTAGGGCTCAACCTTACTGCT-3’)  R: (5’- CTCCAACAGCGTGTGTGTCTGTAG-3’) | | 95°C, 3 min | | | | Ankarklev et al., 2018 |
|  |  |  | 95°C, 20 sec | |  | |  |
|  |  |  | 55°C, 30 sec | | 35x | |  |
|  |  |  | 72 °C, 45 sec | |  | |  |
|  |  |  | 72 °C, 7 min | | | |  |
| Second amplification  Amplicon size: 557 bp | F: (5’-GACAACGCCTCCGTCACTTC-3’)  R: (5’-GACTCCTTGATGGCATACAACG-3’) | | 95°C, 3 min | | | | Ankarklev et al., 2018 |
|  |  |  | 95°C, 20 sec |  | | |  |
|  |  |  | 55°C, 30 sec | 35x | | |  |
|  |  |  | 72 °C, 45 sec |  | | |  |
|  |  |  | 72 °C, 7 min | | | |  |
| ***Giardia*** High cysteine protein HCMP6372 | | | | | | |  |
| First amplification  Amplicon size: 828 bp | F: (5’-GAACTTGTGTCACGCTGAATTAATACAG-3’)  R: (5’-TCGGGAACACAGACGACACCT-3’) | | 95°C, 3 min | | | | Ankarklev et al., 2018 |
|  |  |  | 95°C, 20 sec | |  | |  |
|  |  |  | 55°C, 30 sec | | 35x | |  |
|  |  |  | 72 °C, 45 sec | |  | |  |
|  |  |  | 72 °C, 7 min | | | |  |
| Second amplification  Amplicon size: 640 bp | F: (5’-GGCGGTGAGTGTGTGGAGAC-3’)  R: (5’-CTTCAGAGATGCAAGTACCATTGTTC-3’) | | 95°C, 3 min | | | | Ankarklev et al., 2018 |
|  |  |  | 95°C, 20 sec |  | | |  |
|  |  |  | 55°C, 30 sec | 35x | | |  |
|  |  |  | 72 °C, 45 sec |  | | |  |
|  |  |  | 72 °C, 7 min | | | |  |
| ***Giardia:* 6-phos-phogluconate dehydrogenase (pgd)** | | | | | | | Seabolt et al., 2021  Seabolt et al., 2021 |
| First amplification  Amplicon size: 1028 bp | F: (5’- GGR ATT RTT GCG CAR TCR CTT CC -3’)  R: (5’- CAG AGA TGT TCG YYT ACG AAA C-3’) | | 95°C, 3 min | | | |  |
|  |  |  | 94°C, 45 sec | | |  |  |
|  |  |  | 58°C, 45 sec | | | 35x |  |
|  |  |  | 72 °C, 90 sec | | |  |  |
|  |  |  | 72 °C, 7 min | | | |  |
| Second amplification | F: (5’- GAC TAT AGY TCR CCA ATA GGC -3’)  R: (5’- TTR TAT CTT GCA GKC AGC TGR CA-3’) | | 95°C, 3 min | | | |  |
|  |  |  | 94°C, 45 sec | | |  |  |
|  |  |  | 58°C, 45 sec | | | 35x |  |
|  |  |  | 72 °C, 90 sec | | |  |  |
|  |  |  | 72 °C, 7 min | | | |  |
| *Giardia***: Hypothetical protein** | | | | | | | Seabolt et al., 2021 |
| First amplification  Amplicon size: 1356 bp | F: (5’- GGT TAC YTT TCT AGG TGA YAT ATA -3’)  R: (5’- CTR CAR AAC GGW AGR CTC ARG TC-3’) | | 95°C, 3 min | | | |  |
|  |  |  | 94°C, 45 sec | |  | |  |
|  |  |  | 58°C, 45 sec | | 35x | |  |
|  |  |  | 72 °C, 90 sec | |  | |  |
|  |  |  | 72 °C, 7 min | | | |  |
| Second amplification, | F: (5’- CAG RGT GCC AAA TCT TTA CRC -3’)  R: (5’- CCC GTG AAT ACR CAY AAG CTA T-3’) | | 95°C, 3 min | | | | Seabolt et al., 2021 |
|  |  |  | 94°C, 45 sec |  | | |  |
|  |  |  | 58°C, 45 sec | 35x | | |  |
|  |  |  | 72 °C, 90 sec |  | | |  |
|  |  |  | 72 °C, 7 min | | | |  |
| *Giardia***: Phosphorylase B gamma catalytic chain kinase (phkg2)** | | | | | | | Seabolt et al., 2021 |
| First amplification  Amplicon size: 1017 bp | F: (5’- CTT GAC CTY AAT GCM TTY CTY ATG A -3’)  R: (5’- GCT YTT GTT CTG YCC AAG GCT-3’) | | 95°C, 3 min | | | |  |
|  |  |  | 94°C, 45 sec | |  | |  |
|  |  |  | 58°C, 45 sec | | 35x | |  |
|  |  |  | 72 °C, 90 sec | |  | |  |
|  |  |  | 72 °C, 7 min | | | |  |
| Second amplification | F: (5’- AAT CTG TCC YCT YGA GAT TGC T -3’)  R: (5’- TGA AGA GCC TCC GAG AAR TC-3’) | | 95°C, 3 min | | | | Seabolt et al., 2021 |
|  |  |  | 94°C, 45 sec |  | | |  |
|  |  |  | 58°C, 45 sec | 35x | | |  |
|  |  |  | 72 °C, 90 sec |  | | |  |
|  |  |  | 72 °C, 7 min | | | |  |
| We used the following PCR mixture for all genes: reactions were carried out in a total volume of 25 µL that included 2 µL of template DNA, 1 µL forward and 1 µL reverse primer (0.4µM concentration) and 12.5 µL of DreamTaq PCR Master Mix (2X) (Thermo Fisher Scientific) and 8.5 µL nuclease-free water. 0.2 µL Bovine serum albumin (20mg/ml) was used in the reaction for SSU, BG, TPI, and GDH gene. BSA was not included for *Cryptosporidium* and *Giardia* assemblage A and B subtyping. | | | | | | | |

References

Ankarklev, J., Lebbad, M., Einarsson, E., Franzén, O., Ahola, H., Troell, K., Svärd, S.G., 2018. A novel high-resolution multilocus sequence typing of Giardia intestinalis Assemblage A isolates reveals zoonotic transmission, clonal outbreaks and recombination. Infect. Genet. Evol. 60, 7–16. https://doi.org/10.1016/j.meegid.2018.02.012

Cacciò, S.M., Beck, R., Lalle, M., Marinculic, A., Pozio, E., 2008. Multilocus genotyping of Giardia duodenalis reveals striking differences between assemblages A and B. Int. J. Parasitol. 38, 1523–1531. https://doi.org/10.1016/j.ijpara.2008.04.008

Jiang, J., Alderisio, K.A., Xiao, L., 2005. Distribution of Cryptosporidium genotypes in storm event water samples from three watersheds in New York. Appl. Environ. Microbiol. 71, 4446–4454. https://doi.org/10.1128/AEM.71.8.4446-4454.2005

Lalle, M., Pozio, E., Capelli, G., Bruschi, F., Crotti, D., Cacciò, S.M., 2005. Genetic heterogeneity at the β-giardin locus among human and animal isolates of Giardia duodenalis and identification of potentially zoonotic subgenotypes. Int. J. Parasitol. 35, 207–213. https://doi.org/10.1016/j.ijpara.2004.10.022

Li, N., Xiao, L., Alderisio, K., Elwin, K., Cebelinski, E., Chalmers, R., Santin, M., Fayer, R., Kvac, M., Ryan, U., Sak, B., Stanko, M., Guo, Y., Wang, L., Zhang, L., Cai, J., Roellig, D., Feng, Y., 2014. Subtyping Cryptosporidium ubiquitum, a zoonotic pathogen emerging in humans. Emerg. Infect. Dis. 20, 217–224. https://doi.org/10.3201/eid2002.121797

Read, C., Walters, J., Robertson, I.D., Thompson, R.C.A., 2002. Correlation between genotype of Giardiaduodenalis and diarrhoea. Int. J. Parasitol. 32, 229–231. https://doi.org/10.1016/S0020-7519(01)00340-X

Seabolt, M.H, Konstantinidis, K.T., Roellig, D.M. 2021. Hidden diversity within common protozoan parasites as revealed by a novel genomotyping scheme. Appl Environ. Microbiol 87:e02275-20. https://doi.org/10.1128/AEM.02275-20.

Sulaiman, I.M., Fayer, R., Bern, C., Gilman, R.H., Trout, J.M., Schantz, P.M., Das, P., Lal, A.A., Xiao, L., 2003. Artigo Tpi_Sulaiman_2003. Emerg. Infect. Dis. 9, 15–17.
