# Supplementary material for: Cryptosporidium and Giardia in Livestock in Tigray, Northern Ethiopia and Associated Risk Factors for Infection: A Cross-Sectional Study
Source: Front Vet Sci. 2022 Jan 14;8:825940. doi: 10.3389/fvets.2021.825940 (PMC8795829; doi:10.3389/fvets.2021.825940)
Supplement: Supplementary file 2 [file Data_Sheet_2.docx]

**Questionnaire for calf** **owners**

1**. General information**

**A. Participant**

1.1. Name of the farm and owner_________________ code no._______________

1.2. Date of sampling_______________________

**B. Calves**

| **No** | **Sex** | **Age** | **Breed** | **District** | **Management** | **Faeces consistency** | **Colour** | **Presence / absence of mucus** | **Pen cleanliness** | **Cleanliness** |
| --- | --- | --- | --- | --- | --- | --- | --- | --- | --- | --- |
| **1** |  |  |  |  |  |  |  |  |  |  |
| **2** |  |  |  |  |  |  |  |  |  |  |

**2. Farm Profile and farming experience**

2.1. Number of calves and adult cows on farm.

1. ____calves 2. ______adults

2.2. What is your role in this farm?

1. Hired calf caretaker (attendant)

2. Owner and calf caretaker

3. Family member (child) and calf caretaker

2.3. Could you tell us about periparturient care on the farm?

1. Calves in their pen alone or calves in their pen with other calves

2. Calves stay in the same barn with cow

2.4. Do you feed colostrum to neonates?

1. Yes 2. No

2.5. If your answer is yes, when are calves fed colostrum?

1. Immediately (<3 h after birth) 2. 3 h or more after birth

2.6. What is the weaning age for the calves?

1. > 6 months 2. < 6 months

2.7. When is feed other than milk introduced to calves?

1. 4 weeks 2. 2 weeks 3. 3 weeks 4. other

2.8. How are calves fed?

1. Feed placed in feeding trough 2. Feed placed on ground (no feeding trough)

3. Grazing

2.9. What type of additional feed is provided for your calves?

1. Grazing on pastureland 2. Concentrates 3. Hay

2.10. Do young calves mix with adults on grazing land?

1. Yes 2. No 3. Sometimes

2.11. Where do calves obtain drinking water?

1. Communal drinking place with adults

2. Communal drinking place with other calves

3. Individual drinking place

2.12. What is the source of drinking water for calves?

1. River 2. Ponds 3. Hand pump 4. Well

3. **Cryptosporidiosis and giardiasis in calves**

3.1. Have you ever experienced diarrhoea in calves on your farm?

1. Yes 2.No

3.2. If yes, how often has it happened?

1. Once per month 2. Once in the first six-month period

3.3. Estimate the calf age (in weeks) at which diarrhoea occurs?

1. 1-6wks 2. 7-12 wks 3. 13-18 wks 4.19-24 wks 5. 25-30 wks 6. No

3.4. Which other signs have you observed among young diarrhoeic calves?

1. Unable to suckle or feed

2. Poor weight gain/stunted growth

3. Mortality

3.5. When calves have diarrhoea, how do you clean their house?

1. No special cleaning management

2. Immediately cleaned and the waste material disposed of in a designated disposal area

3. Cleaning immediately, but disposal anywhere on the pasture

3.6. Have you ever noticed droppings/dung contaminating drinking water or feed?

1. Yes 2. No

3.7. How do you treat cases of diarrhoea among calves?

1. No treatment

2. Visit vet clinic

3. Homemade treatment

2. **Questionnaire for lamb and/or kid owners**

1. **General information**

**A. Participant**

1.1. Name of the farm and owner____________________ code no.____________

1.2. Date of sampling______________________________

**B. Lambs**

| **No** | **Sex** | **Age** | **Breed** | **District** | **Management** | **Faeces consistency** | **Colour** | **Presence / absence of mucus** | **Pen cleanliness** | **Cleanliness** |
| --- | --- | --- | --- | --- | --- | --- | --- | --- | --- | --- |
| **1** |  |  |  |  |  |  |  |  |  |  |
| **2** |  |  |  |  |  |  |  |  |  |  |

**C. Kids**

| **No** | **Sex** | **Age** | **Breed** | **District** | **Management** | **Faeces consistency** | **Colour** | **Presence / absence of mucus** | **Pen cleanliness** | **Cleanliness** |
| --- | --- | --- | --- | --- | --- | --- | --- | --- | --- | --- |
| **1** |  |  |  |  |  |  |  |  |  |  |
| **2** |  |  |  |  |  |  |  |  |  |  |

**2. Farm Profile and farming experience**

2.1. Number of lambs, kids and adult sheep/goats on this farm

1. ____Lambs 2. ____Kids 3. ______Adults

2.2. What is your role in this farm?

1. Hired sheep/goat shepherd

2. Owner and caretaker of sheep and goats

3. Family member (child) and caretaker of sheep and goats

2.3. Could you tell us about periparturient care on the farm?

1. Ewes and lambs/does and goat kids in their pen

2. Ewes and lambs/ does and goat kids mixed with other animals

2.4. Do you feed colostrum to neonatal lambs / kids?

1. Yes 2. No

2.5. If your answer is yes, when are lambs/kids fed colostrum?

1. Immediately (<3h after birth) 2. >3 h after birth

2.6. How are lambs / kids fed?

1. Feed placed in the feeding trough

2. Feed placed on the ground (no feeding trough)

3. Grazing

2.7. What type of additional feed is provided for you lambs and kids?

1. Grazing on pastureland 2. Concentrates 3. Hay

2.8. Do lambs and kids graze with adult sheep/goats?

1. Yes 2. No 3. Sometimes

2.9. Where do lambs and kids obtain drinking water?

1. Communal drinking place with adults

2. Communal drinking place with other lambs and kids

3. Individual drinking place

2.10. What is the source of drinking water for lambs/kids?

1. River 2. Ponds 3. Hand pump 4. Well

**3. Cryptosporidiosis and giardiasis in lambs and kids**

3.1. Have you ever experienced diarrhoea in lambs / kids on your farm?

1. Yes 2. No

3.2. If yes, how often has it happened?

1. Once per month 2. Once in the first six-month period

3.3. Estimate the age of the lambs/kids in weeks at which diarrhoea occurs?

1. 1-6wks 2. 6-12wks 3. 12-18wks 4. 18-24wks 5. 24-30wks 6. No

3.4. Which other signs have you observed among young diarrheic lambs/kids?

1. Unable to suckle or feed

2. Poor weight gain/stunted growth

3. Mortality

4. No clinical signs

3.5. When lambs/kids have diarrhoea, how do you clean their house?

1. No special cleaning management

2. Immediately cleaned and the waste material disposed of in a designated area

3. Cleaning immediately, but disposal can be anywhere on the pasture

3.6. Have you ever noticed droppings/dung contaminating drinking water or feed?

1. Yes 2. No

3.7. How do you treat cases of diarrhoea among lambs/kids?

1. No treatment

2. Visit vet clinic

3. Homemade treatment
