# Supplementary material for: Cryptosporidium and Giardia in Livestock in Tigray, Northern Ethiopia and Associated Risk Factors for Infection: A Cross-Sectional Study
Source: Front Vet Sci. 2022 Jan 14;8:825940. doi: 10.3389/fvets.2021.825940 (PMC8795829; doi:10.3389/fvets.2021.825940)
Supplement: Supplementary file 3 [file Data_Sheet_3.docx]

S. Table 1*.* Chi-squared and Fisher’s exact test results on *Cryptosporidium* infection in calves and various risk factors

| **Variables** | **Category** | | **Positives** | **Total samples** | **Proportion (%)** | **P-value** |
| --- | --- | --- | --- | --- | --- | --- |
| Sex | M | | 11 | 119 | 9 | 0.64 |
|  | F | | 10 | 89 | 11 |  |
| Location | Enderta | | 6 | 53 | 11 | 0.03 |
|  | Kilte Awulaelo | | 10 | 48 | 21 |  |
|  | Hintalo Wejirat | | 2 | 49 | 4 |  |
|  | Raya Azebo | | 3 | 58 | 5 |  |
| Periparturient care | Calves in their pen alone or with other calves | | 15 | 183 | 8 | 0.01 |
|  | Calves with the cow | | 6 | 24 | 25 |  |
| Time of colostrum feeding | Immediately (<3hrs) | | 18 | 175 | 10 | 0.59 |
|  | >3 hrs | | 3 | 32 | 9 |  |
| Weaning age | >6 months | | 12 | 175 | 7 | 0.001 |
|  | <6 months | | 9 | 32 | 28 |  |
| Type of additional feed | Pasture | Yes | 6 | 76 | 8 | 0.41 |
|  |  | No | 15 | 131 | 12 |  |
|  | Concentrates | Yes | 13 | 78 | 17 | 0.02 |
|  |  | No | 8 | 129 | 6 |  |
|  | Hay | Yes | 19 | 166 | 12 | 0.26 |
|  |  | No | 2 | 41 | 5 |  |
| Management system | Extensive | | 13 | 173 | 8 | 0.001 |
|  | Semi-intensive | | 1 | 18 | 6 |  |
|  | Intensive | | 7 | 17 | 41 |  |
| Breed | Local | | 13 | 184 | 7 | 0.001 |
|  | Cross | | 8 | 24 | 33 |  |
| **Total** |  | | **21** | **208** | **10** |  |

S. Table 2*.* Chi-squared test results on *Giardia* infection in calves and various risk factors

| **Variables** | **Category** | | **Positives** | **Total samples** | **Proportion (%)** | **P-value** |
| --- | --- | --- | --- | --- | --- | --- |
| Sex | M | | 49 | 119 | 41 | 0.45 |
|  | F | | 32 | 89 | 36 |  |
| Location | Enderta | | 25 | 53 | 47 | 0.16 |
|  | Kilte Awulaelo | | 21 | 48 | 44 |  |
|  | Hintalo Wejirat | | 13 | 49 | 27 |  |
|  | Raya Azebo | | 22 | 58 | 38 |  |
| Periparturient care | Calves in their pen alone or with other calves | | 69 | 183 | 37 | 0.25 |
|  | Calves with the cow | | 12 | 24 | 50 |  |
| Time of colostrum feeding | Immediately (<3hrs) | | 75 | 175 | 43 | 0.01 |
|  | >3 hrs | | 6 | 32 | 19 |  |
| Weaning age | >6 months | | 69 | 175 | 39 | 0.84 |
|  | <6 months | | 12 | 32 | 38 |  |
| Type of additional feed | Pasture | Yes | 25 | 76 | 33 | 0.16 |
|  |  | No | 56 | 131 | 43 |  |
|  | Concentrates | Yes | 37 | 78 | 47 | 0.06 |
|  |  | No | 44 | 129 | 34 |  |
|  | Hay | Yes | 63 | 166 | 38 | 0.48 |
|  |  | No | 18 | 41 | 44 |  |
| Management system | Extensive | | 65 | 173 | 38 | 0.46 |
|  | Semi-intensive | | 7 | 18 | 39 |  |
|  | Intensive | | 9 | 17 | 53 |  |
| Breed | Local | | 68 | 184 | 37 | 0.10 |
|  | Cross | | 13 | 24 | 54 |  |
| **Total** |  | | **81** | **208** | **39** |  |

|  |  |
| --- | --- |

S. Table 3*.* Chi-squared and Fisher’s exact test results on *Cryptosporidium* infection in lambs and various risk factors

| **Variables** | **Category** | | **Positives** | **Total samples** | **Proportion (%)** | **P-value** |
| --- | --- | --- | --- | --- | --- | --- |
| Sex | M | | 10 | 119 | 8 | 0.64 |
|  | F | | 15 | 149 | 10 |  |
| Location | Enderta | | 12 | 73 | 16 | 0.03 |
|  | Kilte Awulaelo | | 3 | 63 | 5 |  |
|  | Hintalo Wejirat | | 3 | 73 | 4 |  |
|  | Raya Azebo | | 7 | 59 | 12 |  |
| Periparturient care | Ewes and lambs in own pen | | 8 | 165 | 5 | 0.002 |
|  | Ewes and lambs together with other animals | | 16 | 101 | 16 |  |
| Time of colostrum feeding | Immediately (<3hrs) | | 20 | 219 | 9 | 0.89 |
|  | >3 hrs | | 4 | 47 | 9 |  |
| Type of additional feed | Pasture | Yes | 16 | 176 | 9 | 0.96 |
|  |  | No | 8 | 90 | 9 |  |
|  | Concentrates | Yes | 2 | 47 | 4 | 0.27 |
|  |  | No | 22 | 219 | 10 |  |
|  | Hay | Yes | 11 | 163 | 7 | 0.10 |
|  |  | No | 13 | 103 | 13 |  |
| Age estimate for diarrhoea occurrence | 1-6 wks | | 3 | 62 | 5 | 0.15 |
|  | 6-12 wks | | 15 | 93 | 16 |  |
|  | 12-18 wks | | 0 | 4 | 0 |  |
|  | 18-24 wks | | 3 | 36 | 8 |  |
|  | 24-30 wks | | 0 | 9 | 0 |  |
|  | No | | 3 | 62 | 5 |  |
| Management system | Extensive | | 15 | 216 | 7 | 0.006 |
|  | Semi-intensive | | 10 | 52 | 19 |  |
| **Total** |  | | **25** | **268** | **9** |  |

S. Table 4*.* Chi-squared and Fisher’s exact test results on *Cryptosporidium* infection in goat kids and various risk factors

| **Variables** | **Category** | | **Positives** | **Total samples** | **Proportion (%)** | **P-value** |
| --- | --- | --- | --- | --- | --- | --- |
| Sex | M | | 6 | 123 | 5 | 0.5 |
|  | F | | 4 | 127 | 3 |  |
| Location | Enderta | | 4 | 67 | 6 | 0.33 |
|  | Kilte Awulaelo | | 3 | 60 | 5 |  |
|  | Hintalo Wejirat | | 0 | 56 | 0 |  |
|  | Raya Azebo | | 3 | 67 | 5 |  |
| Periparturient care | Doe and goat kids in own pen | | 2 | 160 | 1.3 | 0.01 |
|  | Doe and goat kids together with other animals | | 8 | 90 | 9 |  |
| Time of colostrum feeding | Immediately (<3hrs) | | 7 | 214 | 3 | 0.16 |
|  | >3 hrs | | 3 | 36 | 8 |  |
| Type of additional feed | Pasture | Yes | 7 | 193 | 4 | 0.70 |
|  |  | No | 3 | 57 | 5 |  |
|  | Concentrates | Yes | 0 | 28 | 0 | 0.61 |
|  |  | No | 10 | 222 | 5 |  |
|  | Hay | Yes | 4 | 102 | 4 | 0.89 |
|  |  | No | 6 | 148 | 4 |  |
| Age estimate for diarrhoea occurrence | 1-6 wks | | 5 | 67 | 7 | 0.52 |
|  | 6-12 wks | | 3 | 52 | 6 |  |
|  | 12-18 wks | | 0 | 36 | 0 |  |
|  | 18-24 wks | | 0 | 11 | 0 |  |
|  | 24-30 wks | | 0 | 12 | 0 |  |
|  | No | | 2 | 72 | 3 |  |
| Management system | Extensive | | 8 | 223 | 4 | 0.29 |
|  | Semi-intensive | | 2 | 27 | 7 |  |
| **Total** |  | | **10** | **250** | **4** |  |

S. Table 5. Chi-squared and Fisher’s exact test results on *Giardia* infection in lambs and various risk factors

| **Variables** | **Category** | | **Positives** | **Total samples** | **Proportion (%)** | **P-value** |
| --- | --- | --- | --- | --- | --- | --- |
| Sex | M | | 40 | 119 | 34 | 0.63 |
|  | F | | 46 | 149 | 31 |  |
| Location | Enderta | | 32 | 73 | 44 | 0.06 |
|  | Kilte Awulaelo | | 20 | 63 | 32 |  |
|  | Hintalo Wejirat | | 17 | 73 | 23 |  |
|  | Raya Azebo | | 17 | 59 | 29 |  |
| Periparturient care | Ewes and lambs in their pen | | 38 | 165 | 23 | 0.001 |
|  | Ewes and lambs mixed with other animals | | 47 | 101 | 47 |  |
| Time of colostrum feeding | Immediately (<3hrs) | | 69 | 219 | 32 | 0.74 |
|  | >3 hrs | | 16 | 47 | 34 |  |
| Type of additional feed | Pasture | Yes | 53 | 176 | 30 | 0.37 |
|  |  | No | 32 | 90 | 36 |  |
|  | Concentrates | Yes | 19 | 47 | 40 | 0.17 |
|  |  | No | 66 | 219 | 30 |  |
|  | Hay | Yes | 48 | 163 | 29 | 0.27 |
|  |  | No | 37 | 103 | 36 |  |
| Age estimate for diarrhoea occurrence | 1-6 wks | | 13 | 62 | 21 | 0.01 |
|  | 6-12 wks | | 42 | 93 | 45 |  |
|  | 12-18 wks | | 0 | 4 | 0 |  |
|  | 18-24 wks | | 11 | 36 | 31 |  |
|  | 24-30 wks | | 4 | 9 | 44 |  |
|  | No | | 15 | 62 | 24 |  |
| Management system | Extensive | | 63 | 216 | 29 | 0.04 |
|  | Semi-intensive | | 23 | 52 | 44 |  |
| **Total** |  | | **86** | **268** | **32** |  |

S. Table 6*.* Chi-squared and Fisher’s exact test results on *Giardia* infection in goat kids and various risk factors

| **Variables** | **Category** | | **Positives** | **Total samples** | **Proportion (%)** | **P-value** |
| --- | --- | --- | --- | --- | --- | --- |
| Sex | M | | 24 | 123 | 20 | 0.52 |
|  | F | | 29 | 127 | 23 |  |
| Location | Enderta | | 13 | 67 | 19 | 0.27 |
|  | Kilte Awulaelo | | 12 | 60 | 20 |  |
|  | Hintalo Wejirat | | 17 | 56 | 30 |  |
|  | Raya Azebo | | 11 | 67 | 16 |  |
| Periparturient care | Doe and goat kids in their pen | | 30 | 160 | 19 | 0.21 |
|  | Doe and goat kids mixed with other animals | | 23 | 90 | 26 |  |
| Time of colostrum feeding | Immediately (<3hrs) | | 46 | 214 | 22 | 0.78 |
|  | >3 hrs | | 7 | 36 | 19 |  |
| Type of additional feed | Pasture | Yes | 46 | 193 | 24 | 0.06 |
|  |  | No | 7 | 57 | 12 |  |
|  | Concentrates | Yes | 4 | 28 | 14 | 0.46 |
|  |  | No | 49 | 222 | 22 |  |
|  | Hay | Yes | 16 | 102 | 16 | 0.08 |
|  |  | No | 37 | 148 | 25 |  |
| Age estimate for diarrhoea occurrence | 1-6 wks | | 13 | 67 | 19 | 0.35 |
|  | 6-12 wks | | 8 | 52 | 15 |  |
|  | 12-18 wks | | 12 | 36 | 33 |  |
|  | 18-24 wks | | 3 | 11 | 27 |  |
|  | 24-30 wks | | 1 | 12 | 8 |  |
|  | No | | 16 | 72 | 22 |  |
| Management system | Extensive | | 48 | 223 | 22 | 0.72 |
|  | Semi-intensive | | 5 | 27 | 19 |  |
| **Total** |  | | **53** | **250** | **21** |  |
